# Multi-omics insights into *GBA1*-associated Parkinson’s disease: interplay of genomics, transcriptomics, proteomics, and lipidomics

**DOI:** 10.1186/s13024-026-00931-7

**Published:** 2026-01-29

**Authors:** Yang Ni, Huaibin Cai, Yaping Shao, Weidong Le

**Affiliations:** 1 Key Laboratory of Liaoning Province for Research on the Pathogenic Mechanisms of Neurological Diseases, The First Affiliated Hospital, Dalian Medical University, Dalian, China; 2 Transgenic Section, Laboratory of Neurogenetics, National Institute on Aging, National Institutes of Health, Bethesda, MD 20892 USA; 3 Center for Clinical and Translational Medicine, Shanghai University of Medicine and Health Sciences, Shanghai, China

**Keywords:** *GBA1*-associated Parkinson’s disease, Clinical phenotypes, Multi-omics, Metabolic disturbance, Biomarkers

## Abstract

Parkinson’s disease (PD) is the second most prevalent neurodegenerative disorder worldwide. The pathogenesis of PD is driven by multifactorial mechanisms involving a complex interplay among environmental exposures, genetic susceptibility, and aging-related processes. Among genetic contributors, heterozygous pathogenic variants in the *GBA1* gene represent the most significant heritable risk factor for PD. The disease mechanisms of *GBA1* defects in PD remains incompletely understood. It has been proposed that a partial loss-of-function of the lysosomal enzyme glucocerebrosidase, or potential toxic gain-of-function effects (e.g., endoplasmic reticulum stress) might contribute to the disease. These processes initiate a cascade of pathophysiological events, including dysregulated sphingolipid metabolism, compromised lysosomal-autophagic function, mitochondrial dysfunction, and accelerated α-synuclein aggregation. Subsequent dopaminergic neurodegeneration and sustained neuroinflammatory cascades ultimately drive PD progression. Nevertheless, the precise molecular mechanisms linking *GBA1* mutations to PD pathogenesis remain incompletely elucidated, and clinically validated early diagnostic biomarkers for *GBA1*-associated PD (*GBA1*-PD) are still lacking. This review summarizes the distinct clinical phenotypes and mechanistic underpinnings of *GBA1*-PD, with particular emphasis on omics-derived stratification biomarkers (identified through genomics, transcriptomics, proteomics, and lipidomics approaches) coupled with neuroimaging signatures. This review advances our understanding of *GBA1*-mediated PD pathogenesis while providing a framework for developing precision diagnostic strategies and targeted therapeutic interventions addressing PD heterogeneity.

## Background

Parkinson’s disease (PD) exhibits a strong age-dependent prevalence, with annual incidence rates ranging from 108 to 212 per 100,000 individuals aged ≥ 65 years [1]. Neuropathological confirmation of PD requires the postmortem demonstration of two cardinal features: (1) marked degeneration of dopaminergic neurons in the substantia nigra pars compacta, and (2) intraneuronal proteinaceous inclusions composed of fibrillar α-synuclein (α-syn) aggregates, pathologically termed Lewy bodies [1, 2]. In clinical practice, the diagnosis of PD primarily relies on the identification of typical motor manifestations, including asymmetric bradykinesia, rigidity, tremor, and postural instability, while excluding atypical parkinsonian syndromes [3]. PD pathogenesis emerges from the complex interplay among environmental exposures, intrinsic genetic susceptibility and age-related metabolic dysregulation [4]. Advances in high-throughput genomic technologies have identified over 20 monogenic loci and numerous susceptibility variants associated with PD risk [5].

Pathogenic variants in the gene encoding β-glucocerebrosidase (*GBA1*) are the most prevalent genetic risk factor for PD, with approximately 3%-15% of Ashkenazi Jewish patients and 2.98% of Asian-ancestry patients carrying *GBA1* variants [6, 7]. It is noteworthy that homozygous *GBA1* mutations lead to Gaucher disease (GD), while heterozygous mutations significantly increase the risk of developing PD [8]. PD patients carrying *GBA1* pathogenic variants exhibit distinct clinicopathological phenotypes characterized by an earlier disease onset, accelerated progression trajectories, and an increased burden of both motor complications and non-motor comorbidities [9]. Rapid cognitive decline is particularly associated with specific pathogenic variants, notably L444P and N370S [6, 10–12]. Elucidating the pathophysiological mechanisms underlying *GBA1*-associated PD (*GBA1*-PD) is critical for developing personalized therapeutic interventions and optimizing precision medicine paradigms through patient stratification [13].

Heterozygous *GBA1* mutations induce a partial loss-of-function in lysosomal β- glucocerebrosidase (GCase), the principal enzyme responsible for catabolizing glycosphingolipids. This enzymatic deficiency promotes the pathological accumulation of its substrates glucosylceramide (GlcCer) and glucosylsphingosine (GlcSph), along with widespread lipid imbalances [14, 15]. Furthermore, GCase-deficiency-mediated impairment of the lysosomal-autophagic flux can trigger dopaminergic neuronal damage, potentially representing a key pathogenic mechanism in PD development [16, 17]. Nevertheless, the mechanistic interplay between *GBA1* dysfunction and PD pathogenesis remains elusive. Crucially, the current absence of clinically validated, minimally invasive biomarkers confine *GBA1*-PD identification to genetic screening approaches. This limitation highlights an urgent need for pathophysiology-driven biomarker discovery to enable precision subtyping and facilitate presymptomatic detection.

The rapid advancement of multi-omics technologies including genomics, transcriptomics, proteomics, metabolomics, and lipidomics, has fundamentally enhanced our ability to elucidate PD heterogeneity through a systems biology framework. This integrative approach enables the mechanistic stratification of clinical phenotypes while uncovering disease-relevant molecular circuitry, thereby paving the way for biomarker discovery and the development of pathophysiology-informed therapeutic strategies [18]. This review presents a comprehensive integrated analysis of recent multi-omics breakthroughs in *GBA1*-PD pathobiology. We establish multidimensional genotype-phenotype mappings through systematic analysis of *GBA1* variant-specific molecular signatures and summarize emerging biomarker candidates identified via multi-omics techniques. By integrating molecular insights with clinical manifestations, this review establishes a mechanistic foundation for future research into the pathogenic mechanisms underlying *GBA1*-PD and its personalized therapies.

## Genetic architecture of *GBA1*-PD

The *GBA1* gene resides on the long arm of chromosome 1q21 and encompasses approximately 7.6 kb of genomic DNA, which is organized into 11 exons and 10 introns [19]. It encodes GCase (~ 62 kDa), a lysosomal hydrolase essential for hydrolyzing GlcCer into glucose and ceramide (Cer). Notably, a highly homologous pseudogene (*GBA1P*) is positioned ~ 16 kb downstream of *GBA1*, exhibiting 96% exonic sequence identity. While the biological function of *GBA1P* remains uncharacterized, this extensive homology frequently leads to complications in sequencing analyses. To prevent potential misannotation, long-read sequencing or combining long-range PCR with Sanger sequencing is strongly recommended [20].

To date, over 400 *GBA1* variants have been identified globally, including missense variants (e.g., N370S, L444P), nonsense variants (e.g., R359X), splice-site variants (e.g., IVS2 + 1G > A, RecNciI), and other variants (84GG), with missense variants being the most prevalent [21]. Homozygous pathogenic *GBA1* mutations cause GD, an autosomal recessive lysosomal storage disorder characterized by deficient GCase activity. This enzyme deficiency leads to GlcCer accumulation in macrophages of the liver, spleen, bones, bone marrow, and central nervous system, forming pathognomonic Gaucher cells and resulting in multisystem damage [22]. Notably, GD patients and their first-degree relatives show an increased risk of PD [23, 24], and large-scale studies confirm that heterozygous *GBA1* mutations significantly increase PD susceptibility [6, 25]. Over 300 *GBA1* variants have been implicated in PD risk [26, 27], with notable ethnic heterogeneity observed among patients carrying these mutations. Ashkenazi Jewish populations exhibit the highest prevalence (10%-30%), driven predominantly by the N370S variant [6]. In non-Ashkenazi Jewish cohorts, frequencies range from 2.9% to 12%, with L444P being more prevalent in Chinese populations [28–30]. In Europeans, E326K and T369M variants exhibit elevated carrier frequencies [26]. Notably, PD-associated *GBA1* variants cluster in exons 8–10, including key substitutions such as L444P, N370S, E326K, T369M, and D409H [26].

*GBA1* variants are classified into five categories according to their impact on GD severity: “severe” variants (e.g., L444P, IVS2 + 1G > A, 84GG, and D409H) which lead to neuropathic GD (types II/III) and correlate with a higher risk of PD and more severe PD phenotypes; “complex” variants (e.g., RecNciI, RecTL) which represent a severe subtype resulting from homologous recombination between *GBA1* and its pseudogene *GBA1P*, leading to pathogenic multivariant alleles; “mild” variants (e.g., N370S), which lead to non-neuronopathic GD (type I) with a moderate PD risk; “risk” variants (e.g., E326K), which increase PD susceptibility without causing GD; and an increasing number of “unknown” variants that require further study to define their roles. Parlar et al. developed an online platform archiving 371 variants associated with *GBA1*-PD, which facilitates variant classification, thereby providing a valuable resource for future research and clinical trials [27]. The penetrance of *GBA1* mutations is affected by age, genetic and non-genetic factors. Studies indicate that among carriers aged 60–80 years, penetrance typically ranges from 10% to 30%. Notably, severe mutations demonstrate higher penetrance compared to mild variants [31, 32].

## Clinical characteristics of *GBA1*-PD

**Fig. 1:**
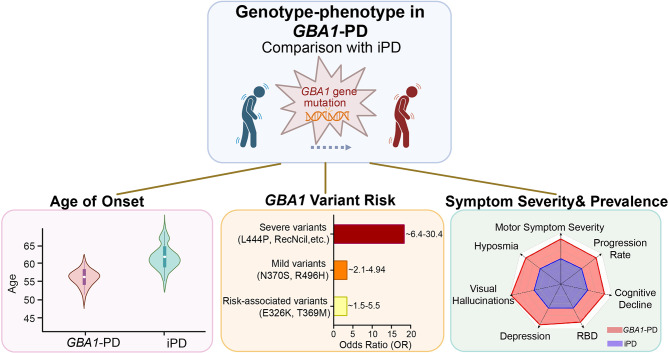
Distinct clinical phenotype of *GBA1*-PD. *GBA1*-PD is characterized by an earlier age of onset and a more aggressive disease progress. The severity of the clinical phenotype is strongly correlated with the specific type of *GBA1* mutation. Patients with *GBA1*-PD typically exhibit a more rapid progression of both motor and non-motor symptoms. This highlights the critical role of *GBA1* genotyping in risk stratification, predicting disease progression, and guiding personalized therapeutic strategies. OR: odds ratios; RBD: rapid eye movement sleep behavior disorder

The phenotypic features of *GBA1*-PD have been extensively characterized. Although there is considerable clinical overlap with idiopathic PD (iPD), which complicates differential diagnosis, *GBA1*-PD exhibits a distinct phenotypic signature (Fig. 1) [5, 30]. The severity of *GBA1* mutations was highly related to the risk of developing PD. Carriers of severe variants (e.g., L444P, RecNciI, 84GG) exhibit significantly elevated odds ratios (ORs = 6.4–30.4). In contrast, mild variants (e.g., N370S, R496H) are associated with a lower risk (ORs = 2.1–4.94), while risk-associated variants (e.g., E326K, T369M) confer intermediate susceptibility (ORs = 1.5–5.5) [27, 33–35]. While the E326K variant is insufficient to cause GD, it has been reported to reduce both the activity and level of GCase in a *GBA1* E326K knock-in mouse model [36]. Moreover, PD patients carrying the E326K variant have a 6.4-fold increased risk of dementia compared to iPD patients, exhibiting significant deficits in working memory, executive function, and visuospatial abilities [37]. A key feature of *GBA1*-PD is an earlier age of onset, occurring on average approximately five years earlier than in non-carriers [33, 38]. The earliest onset is observed in individuals with biallelic *GBA1* mutations (homozygous or compound heterozygous), with a mean age of 56.8 years [35, 39–41]. Furthermore, severe *GBA1* mutations are associated with an average 5-year-earlier onset compared to mild mutations [35]. While both *GBA1*-PD and iPD show a higher prevalence in males, the distribution of mutation types differs by sex. Severe *GBA1* mutations disproportionately affect females, whereas mild mutations and risk variants are more common in males [42].

Clinically, *GBA1* mutations are linked to an accelerated decline in both motor and non-motor functions. Patients with *GBA1*-PD experience a more rapid progression of motor impairments, particularly bradykinesia and axial dysfunction. This decline is most pronounced in carriers of severe variants [11, 43, 44]. Compared to non-carriers, *GBA1*-PD patients exhibit an increased prevalence of rapid eye movement sleep behavior disorder (RBD), autonomic dysfunction, depression, cognitive decline, visual hallucinations, psychiatric symptoms, and more profound hyposmia [39, 45–48]. Moreover, *GBA1* mutations increase susceptibility to dementia with Lewy bodies (DLB) [49–51]. The risk is stratified by mutation severity; specifically, patients with severe mutations exhibit a three-fold higher risk of dementia relative to those with mild mutations, and a five-fold increase compared to PD patients without *GBA1* mutations [44, 51]. *GBA1* mutations are also associated with higher mortality rates. This increased risk is significant for carriers of both severe and mild variants [52, 53]. Furthermore, in the context of advanced therapies like deep brain stimulation, *GBA1*-PD patients tend to derive less postoperative benefit and may experience worse cognitive outcomes, aggravated non-motor symptoms, and a poorer quality of life [54–56]. These distinctions highlight the clinical utility of *GBA1* genotyping for risk stratification, prognostic counseling, and the development of personalized management strategies.

## Mechanistic insights into *GBA1*-PD pathogenesis

**Fig. 2:**
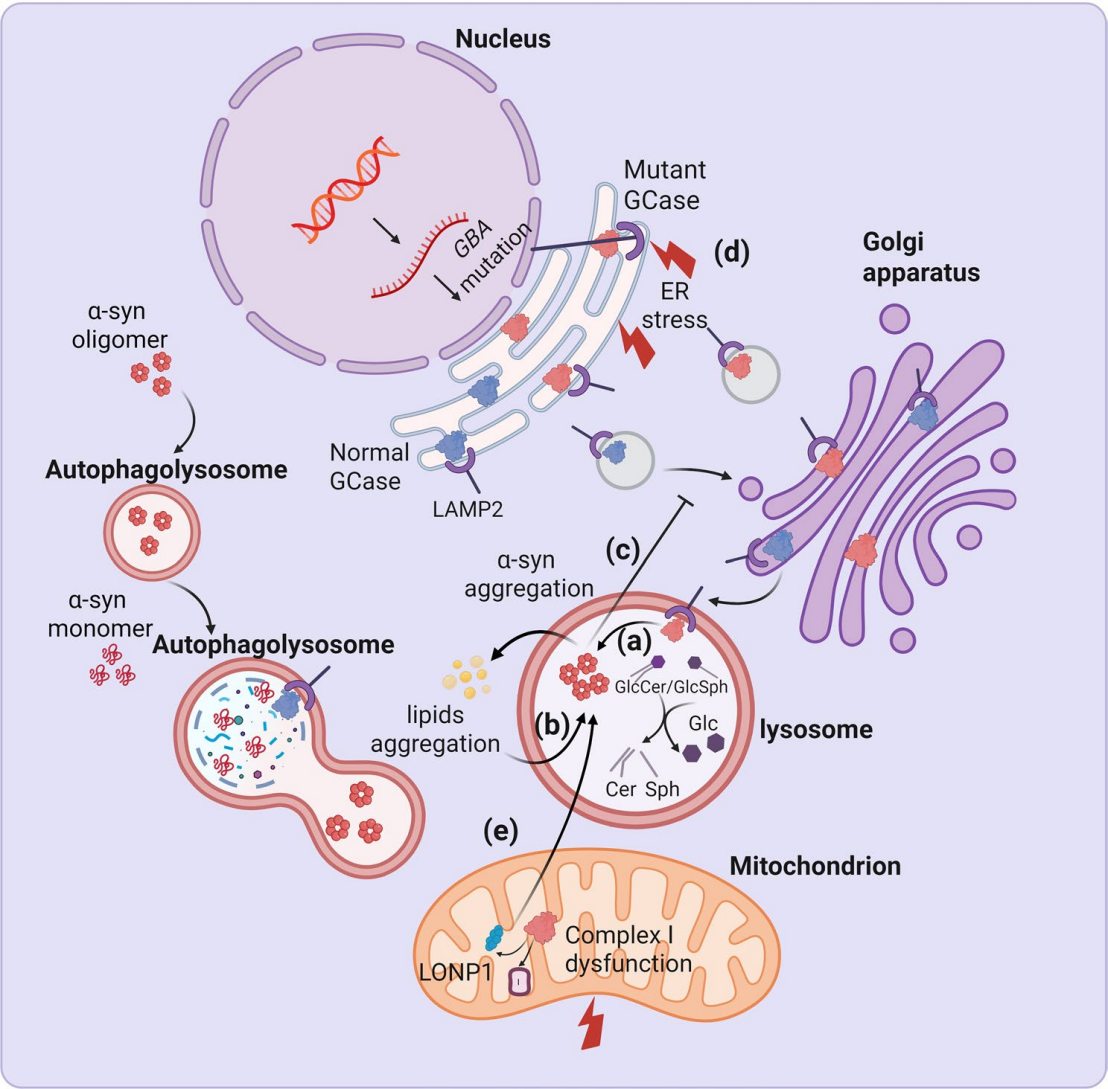
Pathways related to GCase dysfunction in PD. Under physiological conditions, GCase is synthesized in the endoplasmic reticulum (ER). It is then transported to the Golgi apparatus via interaction with the lysosomal integral membrane protein type-2 (LIMP-2), and ultimately targeted to lysosomes. In lysosomes, GCase catalyzes the hydrolysis of glucosylceramide (GlcCer) and glucosylsphingosine (GlcSph) into glucose and ceramide/sphingosine. Typically, α-synuclein (α-syn) is primarily degraded via lysosomal pathways. This process relies on normal lysosomal function, which is critically maintained by GCase activity. In the presence of *GBA1* mutations, several pathological mechanisms are triggered: (**a**) reduced GCase activity leads to impaired lysosomal function, which in turn compromises α-syn degradation and promotes its accumulation; (**b**) accumulation of lipid substrates (GlcCer/GlcSph) exacerbates pathological α-syn aggregation; (**c**) α-syn aggregates block GCase trafficking from the ER to the Golgi; (**d**) misfolded GCase accumulates in the ER, thereby triggering ER stress; and (**e**) misfolded GCase suppresses mitophagy, while inducing mitochondrial oxidative stress and promoting α-syn accumulation by impairing mitochondrial Complex I integrity and disrupting the interaction with LONP1. GCase: β- glucocerebrosidase; ER: endoplasmic reticulum; GlcCer: glucosylceramide; GlcSph: glucosylsphingosine; Cer: ceramide; Sph: sphingosine; LAMP2: lysosome-associated membrane protein 2; LONP1: LON protease homolog, mitochondrial

The functions of GCase are not limited to regulating lipid metabolism; it is also a key regulatory protein within the autophagy-lysosomal pathway (ALP), an essential intracellular system for waste degradation and recycling. The prevailing hypothesis for the core pathogenesis of *GBA1*-PD is the bidirectional positive feedback loop between GCase dysfunction and α-syn aggregation (Fig. 2) [57]. The loss-of-function hypothesis proposes that *GBA1* mutations disrupt GCase structure and function, leading to reduced enzymatic activity. The most direct biochemical consequence of reduced GCase activity is the accumulation of its substrates, primarily GlcCer and GlcSph, within lysosomes and other organelles, which can alter the physicochemical properties of cellular membranes, such as fluidity and curvature. This altered lipid environment has been demonstrated to selectively stabilize toxic α-syn oligomers, thereby promoting their conversion into fibrillar aggregates and facilitating intercellular propagation, which further exacerbates pathological α-syn aggregation [58, 59]. Furthermore, the progressive accumulation of α-syn oligomers and aggregates can block GCase trafficking from the endoplasmic reticulum (ER) to the Golgi apparatus, leading to further lysosomal GCase depletion and creating a pathological vicious cycle [58]. This concept is supported by neuropathological findings in *GBA1*-PD patients, which reveal greater co-localization of GCase within Lewy bodies compared to iPD cases [60], providing direct evidence of the close interplay between these proteins in the pathological state.

However, reduced GCase activity alone is insufficient to fully explain the pathogenesis of *GBA1*-PD. Epidemiological studies indicate that fewer than 10% of GD patients develop PD before the age of 80, suggesting that the vast majority remain unaffected [61]. This indicates the presence of complex and multifactorial interactions that extend beyond simple GCase deficiency [62]. The gain-of-function hypothesis suggests that *GBA1* mutations lead to the accumulation of misfolded GCase within the ER, triggering ER stress and activating the unfolded protein response (UPR). Sustained UPR activation acts as a pro-apoptotic signal, exerting direct neurotoxicity and promoting neurodegeneration [63]. A recent study demonstrated that misfolded GCase impairs α-syn degradation by inhibiting chaperone-mediated autophagy [64]. Additionally, emerging transcriptomic evidence suggests that *GBA1* mutations may upregulate *SNCA* (the gene encoding α-syn), thereby elevating its baseline synthesis [65]. Thus, this vicious cycle, characterized by a concurrent increase in α-syn production and the impairment of its clearance, may drive the earlier onset and more aggressive disease trajectory that distinguish *GBA1*-PD from iPD [9].

Furthermore, these core pathological processes trigger a cascade of downstream molecular events, most notably mitochondrial dysfunction [66]. For instance, studies in *GBA1*^L444P/+^ mice demonstrate that *GBA1* mutations disrupt mitochondrial function via suppressed mitophagy and increased oxidative stress, a mechanism that has also been observed in the anterior cingulate cortex of *GBA1*-PD patients [67]. Furthermore, a recent study has shown that mutant GCase can destabilize mitochondrial complex I, consequently impairing energy metabolism. Concurrently, the mutant GCase enhances its interactions with the mitochondrial protease LONP1, which accelerates the enzyme’s degradation within mitochondria and promotes α-syn aggregation [66].

**Fig. 3:**
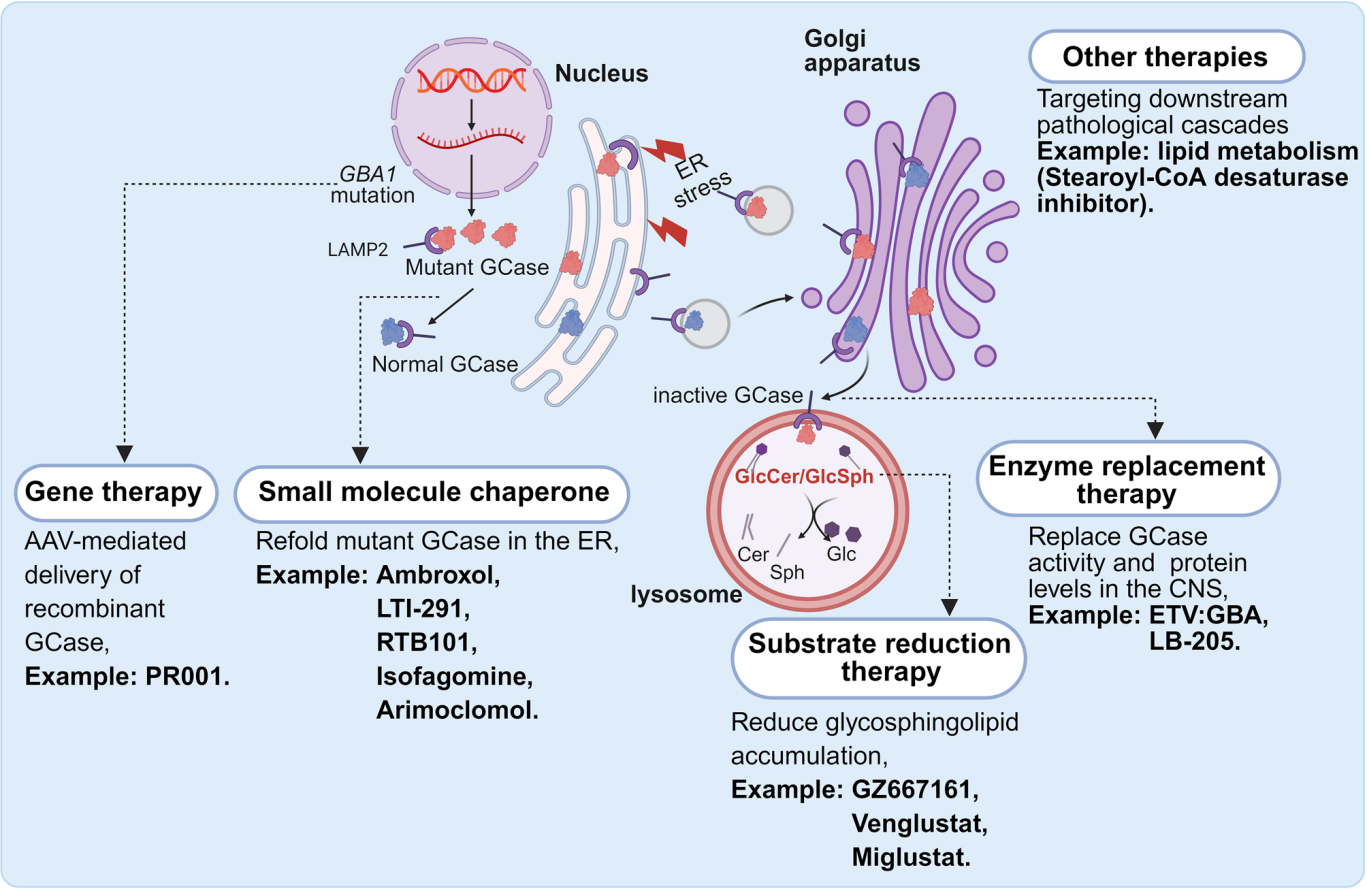
Therapeutic strategies for *GBA1*-PD. Gene therapy aims to deliver a healthy, functional copy of the *GBA1* gene to the cells of the central nervous system to restore GCase production. Small molecule chaperones, such as Ambroxol, are designed to bind to misfolded mutant GCase, stabilizing its conformation and facilitating its proper trafficking from the ER to the lysosome, thereby increasing its activity. Substrate reduction therapy aims to reduce the metabolic burden on the cell by inhibiting glucosylceramide synthase. Enzyme replacement therapy delivers functional recombinant enzymes to lysosomes, substituting deficient endogenous enzymes to restore metabolic function. In addition to GCase-centric approaches, therapies are being developed to target the downstream consequences of *GBA1*-PD pathology, such as monounsaturated fatty acid metabolism. LAMP2: lysosome-associated membrane protein 2; GCase: β-glucocerebrosidase; ER: endoplasmic reticulum; AAV: ‌adeno-associated virus

Investigations into the pathogenesis of *GBA1*-PD have led to diverse therapeutic strategies, which have been reviewed elsewhere (Fig. 3) [13, 68]. While these strategies primarily focus on restoring the enzymatic activity of GCase and reducing pathogenic glycosphingolipid substrates, recent research has uncovered novel molecular pathways. For instance, a recent study revealed that cultured neurons from *GBA1*-PD patients exhibit elevated levels of stearoyl-CoA desaturase (SCD) products (monounsaturated fatty acids) and a concomitant decrease in the α-syn tetramer-to-monomer ratio [69]. Notably, the inhibition of SCD normalized GCase maturation and reduced the accumulation of lysosomal and lipid-rich aggregates, highlighting SCD as a promising novel therapeutic target (Fig. 3). Therefore, leveraging multi-omics approaches including transcriptomics, proteomics, and metabolomics, is a promising strategy to elucidate complex biological pathways and identify novel therapeutic avenues for *GBA1*-PD.

## Neuroimaging features of *GBA1*-PD

Neuroimaging represents a critical translational modality for deciphering genotype-phenotype convergence in neurodegenerative disorders. By revealing multilayered abnormalities in brain structure, functional networks, and molecular pathways, it provides complementary evidence to genetic and clinical findings.

Structural magnetic resonance imaging (MRI) studies suggest a more accelerated and distinct pattern of neurodegeneration in *GBA1*-PD. Voxel-based morphometry studies, although often limited by small sample sizes, have provided evidence of faster rates of cortical thinning in *GBA1*-PD patients compared to non-carriers. This atrophy appears to be most prominent in the temporal, parietal, and occipital regions, a finding that aligns with the accelerated motor and cognitive decline observed in these patients [70]. Additionally, distinct patterns of cortical thickness and serum neurofilament light chain levels have been observed across different *GBA1* mutation subtypes [71]. Furthermore, this cortical vulnerability is corroborated by diffusion tensor imaging (DTI), a technique that assesses the microstructure of white matter (WM) tracts. DTI studies have demonstrated significant WM microstructural alterations in *GBA1*-PD compared to iPD patients. These alterations include reduced fiber-bundle cross section and decreased fiber density and cross section (FDC) within the corticospinal tract, middle cerebellar peduncle, and striato-thalamo-cortical pathways. Critically, these reduced FDC values correlate with lower GCase activity, poorer cognitive performance, and higher motor symptom severity [72]. These findings suggest that the pathology in *GBA1*-PD not only affects gray matter neurons but also compromises the structural connectivity between brain regions [73].

Compared to iPD patients, those with *GBA1*-PD exhibit a distinct functional network reorganization. This is characterized by increased nodal centrality in key regions of the default mode network, specifically the medial prefrontal cortex and the precuneus, along with changes in the sensorimotor cortex. Notably, the increased centrality in the medial prefrontal cortex is positively correlated with fatigue severity [74]. Furthermore, compared to non-carriers, *GBA1*-PD patients exhibit reduced functional connectivity between striatal structures and parieto-occipital cortical areas, a pattern of network dysfunction reminiscent of that observed in patients with DLB and PD dementia. These consistent functional alterations represent promising candidate biomarkers for monitoring disease progression in this high-risk population. Longitudinal studies are warranted to determine whether these network features can reliably predict the onset and trajectory of cognitive decline in *GBA1*-PD [75].

Molecular imaging techniques, such as single-photon emission computed tomography (SPECT) with tracers like ^123^I-ioflupane and positron emission tomography (PET) with tracers like [^18^F]-DOPA are the gold standard for quantifying the loss of presynaptic dopamine terminals in the nigrostriatal pathway. In line with the more aggressive motor phenotype of *GBA1*-PD, a diminished dopamine synthesis capacity in the bilateral caudate nuclei, ipsilateral antero-medial putamen, and contralateral nucleus accumbens was observed in *GBA1*-PD patients [75]. A carefully matched case-control study using quantitative dopamine transporter (DAT) SPECT analysis identified significantly lower specific binding ratios in the most affected anterior putamen and the left caudate of *GBA1*-PD patients compared to iPD controls [76]. This suggests a potentially distinct spatial pattern of denervation. However, upon re-analysis with rigorous correction for multiple comparisons, these differences were no longer statistically significant, highlighting the need for future larger-scale studies. Another study demonstrated significantly lower standardized uptake value ratios (SUVRs) in the ventral striatal and extra-striatal regions of early-stage *GBA1*-PD patients [77]. Longitudinal data indicated accelerated DAT loss in the iPD cohort over a two-year follow-up period, with values converging toward those of *GBA1*-PD patients, suggesting that *GBA1* mutations drive an early phase of neurodegeneration [77]. Additionally, reduced SUVRs in the bilateral parietal and selective occipital cortices were also observed in *GBA1*-PD patients via [^18^F] FP-CIT PET scan. This pattern of parieto-occipital hypoperfusion was consistent with observations in DLB and implicated widespread cortical α-syn pathology [78].

The pathology of PD extends beyond the brain, with systemic manifestations detectable by imaging techniques. Cardiac scintigraphy using the norepinephrine analog ¹²³I-metaiodobenzylguanidine revealed a reduced heart-to-mediastinum ratio in *GBA1*-PD, demonstrating cardiac sympathetic denervation and confirming peripheral autonomic nervous system dysfunction in these patients [78]. Additionally, a recent study using PET with the vesicular acetylcholine transporter ligand [^18^F] FEOBV revealed that patients with *GBA1*-PD exhibit more widespread cholinergic neurodegeneration than non-carriers, further supporting the evidence for an accelerated rate of cognitive and motor decline in this subtype [79].

## Transcriptomic signatures of *GBA1*-PD

**Fig. 4:**
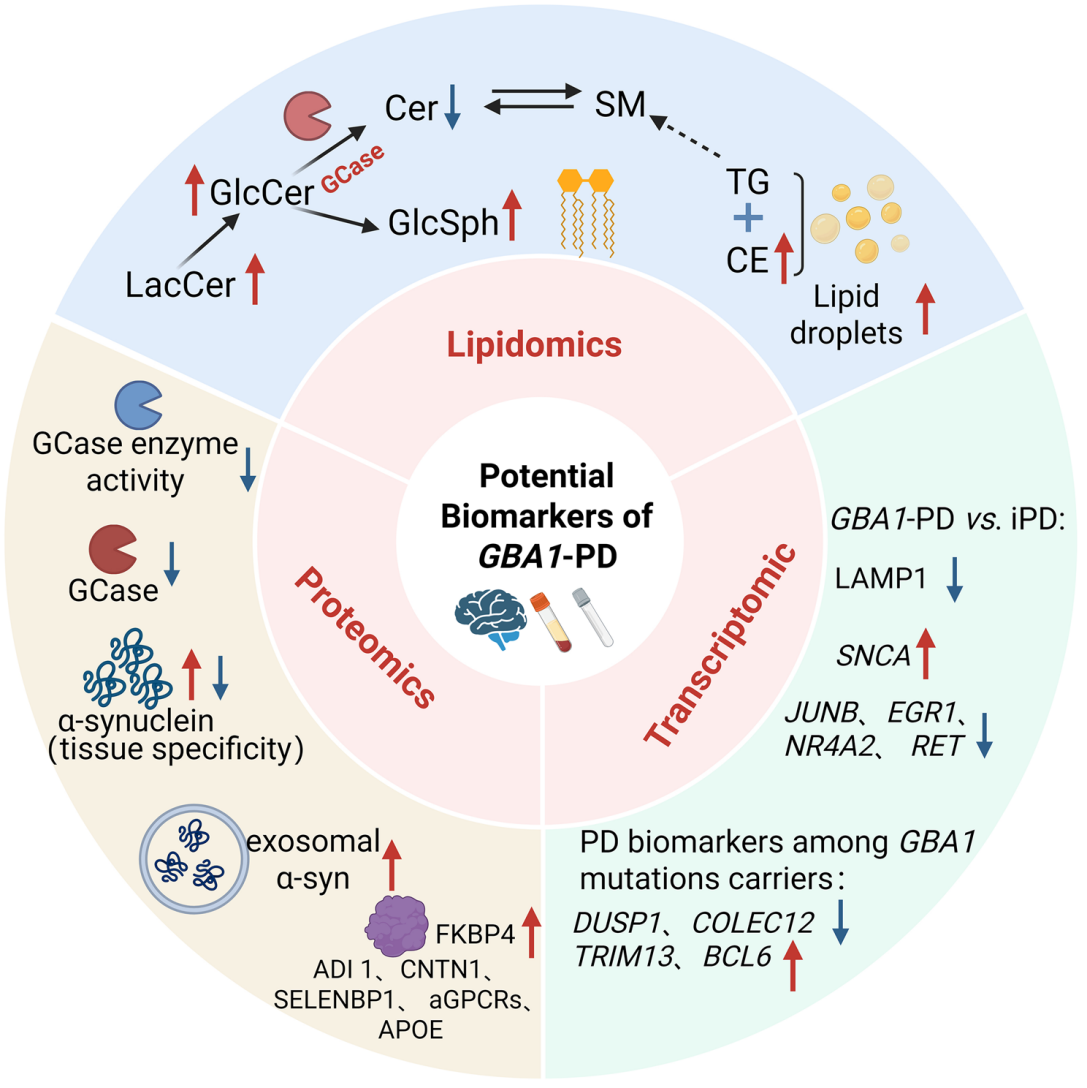
Potential biomarkers and dysregulated molecular pathways of *GBA1*-PD derived from transcriptomics, proteomics and lipidomics studies. This figure highlights the most significant multi-omics findings from peripheral and central tissues, linking them to underlying pathways and potential biomarker applications. LacCer: lactosylceramide; GlcCer: glucosylceramide; GlcSph: glucosylsphingosine; Cer: ceramide; SM: sphingomyelin; TG: triglyceride; CE: cholesterol ester; *GBA1*-PD: *GBA1*-associated Parkinson’s disease; iPD: idiopathic PD; LAMP2: lysosome-associated membrane protein 2; SNCA: synuclein alpha; JUNB: junB proto-oncogene; EGR1: early growth response protein 1; NR4A2: nuclear receptor subfamily 4, group A, member 2; RET: RET proto-oncogene; DUSP1: dual-specificity phosphatase 1; COLEC12: collectin subfamily member 12; TRIM13: Tripartite Motif Containing 13; BCL6: B-cell CLL/lymphoma 6 (zinc finger protein 51)‌; FKBP4: FK506 binding protein 4; ADI1: acireductone dioxygenase 1; CNTN1: Contactin-1; SELENBP1: selenium-binding protein 1; aGPCRs: adhesion G protein-coupled receptors; APOE: apolipoprotein E

Recent transcriptomic studies utilizing patient samples and induced pluripotent stem cell (iPSC)-derived midbrain organoids carrying heterozygous *GBA1* N370S mutations have revealed extensive dysregulation of gene expression networks relevant to *GBA1*-PD [65, 80, 81]. Through the analysis of gene expression profiles in peripheral blood mononuclear cells (PBMCs), whole blood, and iPSCs, researchers have identified potential biomarkers and dysregulated molecular pathways associated with the disease (Fig. 4).

*GBA1* mutations are known to cause aberrant inflammatory responses in monocytes and macrophages. Transcriptomic profiling of isolated CD14 + monocytes has revealed a distinct molecular signature in *GBA1*-PD patients [65]. Compared to iPD, monocytes from *GBA1*-PD patients exhibit significant upregulation of the α-syn gene (*SNCA*), along with other genes involved in its processing and metabolism (e.g., *POLR2D* and *NFATC3*) [65]. These findings suggest that the accumulation of α-syn driven by *GBA1* mutations is regulated by complex mechanisms, extending beyond impaired lysosomal clearance to potentially include increased *SNCA* transcription. Additionally, *GBA1*-PD monocytes also show dysregulation of aging-related genes such as *LMNA* (the gene responsible for progeria syndrome), and amyloid processing pathways (e.g., *ITM2B* and *NCSTN*), suggesting a broader pro-degenerative cellular environment [65].

Comparative transcriptomics in monocyte-derived macrophages ​​from PD patients carrying the L444P mutation​​ identified dysregulated zinc metabolism pathways (e.g., *MT1F*,* MT1X* and *SLC39A8*), indicating that disrupted intracellular zinc homeostasis may exacerbate neurodegeneration through oxidative stress and impaired innate immunity [81]. In parallel, studies of iPSC-derived dopaminergic neurons have shown that the E326K mutation leads to transcriptomic disruptions in ​extracellular matrix receptor interactions, focal adhesion, and PI3K-Akt signaling​​. These pathways point to a dysregulated​​ neuronal microenvironment that contributes to synaptic deficits and impaired cell survival [82]. Furthermore, studies have reported decreased expression of genes critical for dopaminergic neuron identity, including *JUNB*, *EGR1* and *NR4A2*. This molecular change is associated with neuronal loss and reduced dendritic complexity, indicating a disruption of dopaminergic neuronal homeostasis in *GBA1*-PD [80, 81]. It is worth noting that the downregulation of *JUNB* appears to be associated with the presence of a *GBA1* mutation, irrespective of the clinical status.

While peripheral transcriptomics is valuable, RNA-sequencing of post-mortem brain tissue provides the most direct insight into the molecular pathology at the site of neurodegeneration. However, most large-scale transcriptomic studies of PD brains have not specifically stratified samples by *GBA1* status. Therefore, *GBA1*-PD-specific brain transcriptomic signatures have yet to be fully elucidated. A single-nucleus RNA sequencing study of cortical tissue from *GBA1* mutant and *GBA1-SNCA* double-mutant mice revealed a robust downregulation of a gene network regulating synaptic vesicle cycling and synapse assembly. This transcriptional alteration occurred specifically in excitatory neurons, providing a clear molecular basis for the synaptic dysfunction and cognitive deficits observed in *GBA1*-PD [83].

## Protein characteristics of *GBA1*-PD

### GCase enzyme activity

Dysfunction of the lysosomal enzyme GCase, caused by *GBA1* mutations, is strongly implicated in the pathogenesis of *GBA1*-PD [84]. This enzymatic deficiency is evident in both the peripheral and central nervous systems, with significantly reduced GCase activity observed in the blood and cerebrospinal fluid (CSF) of *GBA1*-PD patients [84–88]. Crucially, this enzymatic defect has also been observed in brain tissue, particularly within the substantia nigra of *GBA1*-PD patients [89]. Strikingly, reduced GCase activity and protein levels have also been found in the substantia nigra and cerebellum of PD patients without *GBA1* mutations [89, 90]. This finding suggests that GCase dysfunction is not merely a feature of a genetic subtype but may represent a core pathogenic mechanism in the broader PD population.

The accessibility and minimally invasive nature of peripheral blood samples make them an attractive alternative to brain tissue or CSF for biomarker discovery. Measuring GCase activity in peripheral blood leukocytes or cultured skin fibroblasts is already the gold standard for diagnosing GD [91]. This principle has been extended to PD, with studies confirming that GCase activity is significantly reduced in the blood of iPD patients, which is in line with the enzymatic deficits observed in brain tissue [84]. However, this measurement is complicated by significant inter-cellular heterogeneity. For instance, GCase activity is markedly reduced in monocytes from PD patients compared to controls, whereas no such difference is observed in lymphocytes [92].

### α-synuclein related proteins

The pathological aggregation of α-syn into Lewy bodies is a central feature of PD, making it a key target for biomarker development. However, its utility as a reliable biomarker in patients with *GBA1*-PD remains equivocal due to inconsistent findings across different studies.

Neuropathological investigations have demonstrated that *GBA1*-PD patients exhibit enhanced α-syn deposition in the substantia nigra [90] and significantly increased levels of phosphorylated α-syn (pS129-α-syn) in the cingulate cortex [93]. However, there are no significant differences in cortical Lewy body density when compared to iPD patients [94]. This inconsistency extends to peripheral biomarkers. Analyses of CSF show that total α-syn levels are reduced in *GBA1*-PD and progressively decrease with increasing disease severity [85]. In contrast, α-syn profiles in PBMCs can effectively discriminate *GBA1*-PD from iPD, yet measurements in plasma and erythrocytes lack diagnostic utility [95, 96]. However, plasma levels of oligomeric α-syn are significantly higher in *GBA1*-PD patients compared to controls [97]. These discrepancies likely arise from differences in sample types and detection methods, underscoring that α-syn, whether measured in tissue, CSF, or plasma, does not yet serve as a reliable biomarker for *GBA1*-PD.

Given the limitations of direct α-syn measurement, research has increasingly focused on the mechanisms of its pathological propagation, particularly through extracellular vesicles (EVs) such as exosomes. Exosomes are small EVs (30–150 nm in diameter) that are released into the extracellular space and function as carriers for bioactive molecules, including proteins like α-syn [98]. In the context of PD, EVs are implicated in the misfolding, aggregation, and cell-to-cell transmission of pathological α-syn [99]. Notably, exosome-associated α-syn oligomers are more efficiently taken up by recipient cells and exhibit greater neurotoxicity [100]. The *GBA1* pathway is directly involved in this process. Deficiency of the GCase enzyme, which results from *GBA1* mutations, has been shown to promote exosome release and significantly increase the abundance of exosome-associated α-syn oligomers [101, 102]. This suggests a compelling mechanism whereby *GBA1* mutations may accelerate the spread of pathological α-syn by enhancing its exosome-mediated secretion [103, 104].

### Inflammation-associated proteins

Mutations in the *GBA1* gene are strongly linked to inflammatory dysregulation in PD. Evidence suggests that patients with *GBA1*-PD exhibit a pro-inflammatory state, characterized by significantly lower lymphocyte counts, higher neutrophil-to-lymphocyte ratios [105], and elevated plasma levels of cytokines such as interleukin-8 (IL-8), monocyte chemoattractant protein-1, and macrophage inflammatory protein-1α [38]. Notably, higher IL-8 levels in these patients correlate with poorer cognitive performance. However, recent studies have found comparable levels of inflammatory markers in both the CSF and peripheral blood when comparing *GBA1*-PD patients to non-carrier controls [106]. Consequently, a clear, inflammation-related biomarker signature for *GBA1*-PD has not yet been defined. While lymphopenia may be a consistent underlying phenotype, further research is needed to determine the precise role of systemic and central inflammatory responses in the specific pathogenesis of *GBA1*-PD.

### Other proteins identified by proteomics

High-throughput proteomics offers great potential for uncovering the molecular mechanisms of *GBA1*-PD and discovering novel biomarkers [107]. A proteomic analysis across five brain regions highlighted a more severe impairment of mitochondrial oxidative phosphorylation in *GBA1*-PD patients compared to those with iPD [90]. In the same study, levels of GCase were significantly reduced. Interestingly, levels of acireductone dioxygenase 1 (ADI1), a protein that may be implicated in autophagy were lowest in iPD but remained comparable to controls in *GBA1*-PD, suggesting it may play a role in a distinct pathological pathway [90]. These findings are also reflected at the cellular level. Proteomics analysis of dopaminergic neurons derived from *GBA1*-PD patients revealed abnormal mTOR signaling pathway activation, defective neurite outgrowth, and impaired mitochondrial motility as key phenotypic features [108]. This provides a mechanistic basis for the mitochondrial deficits observed in brain tissue.

Complementing these mechanistic studies, proteomic analysis of biofluids such as CSF and urine has identified several promising biomarker candidates. One study using SWATH-mass spectrometry on CSF identified significant dysregulation of Contactin-1 (CNTN1), selenium-binding protein 1 (SELENBP1), adhesion G protein-coupled receptors (aGPCRs), and apolipoprotein E (APOE), indicating synergistic effects of synaptic damage, oxidative stress, neuronal loss, and α-syn aggregation in *GBA1*-PD [109]. Furthermore, proteomic analysis of CSF secretory proteins in *GBA1*-PD patients also identified a significant increase in FKBP4, a protein that regulates α-syn-induced immune responses and modulates steroid receptor activity [110].

## Lipidomics hallmarks in *GBA1*-PD

Dysregulation of lipid metabolism is a recognized hallmark of PD pathology [111]. Given that the GCase enzyme is a pivotal lipid metabolism enzyme, understanding the impact of *GBA1* mutations on lipid pathways is crucial for elucidating disease mechanisms. GCase dysfunction resulting from *GBA1* mutations leads to the primary accumulation of its substrate, GlcCer. This initial disruption triggers a cascade of further imbalances, affecting precursor and related substrates such as GlcSph and lactosylceramide (LacCer), as well as downstream sphingolipid intermediates. In this section, we summarize the key lipid metabolic disruptions associated with *GBA1*-PD, with further details provided in Table 1.

**Table 1:** Lipidomic profiling and associated lipid alterations in *GBA1*-PD patients

Analytical platform	Subjects	Sample type	GCase activity	α-syn	GlcCer	GlcSph	Cer	Other lipids	Reference
LC-MS/MS	*GBA1*-PD/PDD/DLB (*n* = 28) iPD/PDD/DLB (*n* = 37) LRKK2-PD/PDD (*n* = 7) Control (*n* = 19)	cingulate cortex frontal cortex putamen cerebellum	↓	↑(p-α-syn)	↑(*GBA1*-PDD, frontal cortex)	↑	-	-	Leyns et al. [93]
LC-MS	*GBA1*-PD (*n* = 6) iPD (*n* = 9) PD-DLB + *GBA1* (*n* = 10) Control (*n* = 15)	Frontal cortex putamen substantia nigra	↓	↑	-	↑	-	-	Gündner et al. [112]
LC-ESI-MS/MS	*GBA1*-PD (*n* = 21) iPD (*n* = 21) Control (*n* = 40)	striatum occipital cortex middle temporal gyrus cingulate gyrus	-	-	↑(middle temporal gyrus) ↑ganglioside	-	-	-	Blumenreich et al. [113]
LC-MS/MS	TUEPAC-MIGAP: *GBA1*-PD (*n* = 102) iPD (*n* = 414) PPMI: *GBA1*-PD (*n* = 36) iPD (*n* = 378) Control (*n* = 183)	CSF	↓	↓	↑	-	ns	LacCer ns ↑SM ↑S1P ↑sph	Lerche et al. [114]
LC-MS	*GBA1*-PD (*n* = 29) PD (LRRK2, *n* = 35) iPD (*n* = 17)	serum	-	-	↑HexCer (GlcCer/ GalCer)	-	↑	↑SM	Guedes et al. [14]
LC–MS/MS	*GBA1*-PD (*n* = 8) iPD (*n* = 8) control (*n* = 8)	plasma	-	-	↑(C16:0, C18:0, C22:0, C24:0, C24:1)	↑	-	↑LacCer (C18:0,C22:0,C22:1)	den Heijer et al. [15]
		CSF	-	-	↑(C20:0)	ns	-	↓LacCer (C18:0,C20:0)	
		PBMC	↓	-	ns	ns	-	ns	
UPLC-MS/MS	*GBA1*-PD (*n* = 20, N370S) *GBA1*-Control (*n* = 20, N370S) iPD (*n* = 20) Control (*n* = 20)	plasma	-	-	ns	↑	ns		Surface et al. [115]

LC-MS: Liquid chromatography- mass spectrometry; ESI: electrospray ionization; UPLC: ultra-high performance liquid chromatography; *GBA1*-PD: *GBA1*-associated Parkinson’s disease; *GBA1*-Control: non-manifesting subjects with *GBA1* pathogenic variants; iPD: idiopathic Parkinson’s disease; DLB: dementia with Lewy bodies; PDD: Parkinson’s disease dementia; LacCer: lactosylceramide; SM: sphingomyelin; GlcCer: glucosylceramide; GlcSph: glucosylsphingosine; Cer: ceramide; S1P: sphingosine-1-phosphate; CSF: cerebrospinal fluid; PBMC: peripheral blood mononuclear cell

**Table 2:** Transcriptomic profiling and associated dysregulated pathways in *GBA1*-PD patients

Genes	Group	Pathway	Sample type	Reference
*SNCA*, *POLR2D*, *NFATC3*	*GBA1*-PD (*n* = 23) *GBA1*-Control (*n* = 13) iPD (*n* = 56) Control (*n* = 66)	alpha-synuclein gene and related genes	monocytes	Riboldi et al. [65]
*LMNA*, *FUCA2*, *HEXB*		aging		
*ITM2B*, *NCSTN*		amyloid pathways		
*LAMTOR2*, *RPTOR*		lysosomal function-mTOR signaling pathway		
*DUSP1*, *TRIM13*	*GBA1*-PD (*n* = 5) *GBA1*-Control (*n* = 4) Control (*n* = 4)	autophagy and in the ubiquitin-proteasome pathway	monocyte-derived macrophages	Usenko T et al. [81]
*COLEC12*, *BCL6*		defense response		
*MT1F*, *MT1X*, *SLC39A8*		dysregulated zinc metabolism pathways		
*JUNB*, *EGR1*, *NR4A2*		dopaminergic neuron function, neuronal differentiation, and neurogenesis		
*FOXA2*, *SOX2*	*GBA1*-PD (*n* = 2) Control (*n* = 2)	signaling pathways regulating pluripotency of stem cells	iPSC-derived midbrain organoids	Rosety I et al. [80]

*GBA1*-PD: *GBA1*-associated Parkinson’s disease; *GBA1*-Control: non-manifesting subjects with *GBA1* pathogenic variants; iPD: Idiopathic PD; *SNCA*: synuclein alpha; P*OLR2D*: RNA polymerase II subunit D; *NFATC*3: Nuclear Factor of Activated T-cells, cytoplasmic 3; *LMNA*: Lamin A/C; F*UCA2*: Alpha-L-Fucosidase 2; *HEXB*: Hexosaminidase Subunit Beta; *ITM2B*: Integral membrane protein 2B; *NCSTN*: Nicastrin; *LAMTOR2*: Late Endosomal/Lysosomal Adaptor, MAPK and MTOR Activator 2; *RPTOR*: Regulatory associated protein of MTOR complex 1; *DUSP1*: dual-specificity phosphatase 1; *TRIM13*: Tripartite Motif Containing 13; *COLEC12*: collectin subfamily member 12; *BCL6*: B-cell CLL/lymphoma 6 (zinc finger protein 51)‌; *JUNB*: junB proto-oncogene; *EGR1*: early growth response protein 1; *NR4A2*: nuclear receptor subfamily 4, group A, member 2; *FOXA2*: forkhead box protein A2; *SOX2*: transcription factor SOX2 (SOX group B)

### GlcCer and GlcSph

The abnormal accumulation of GlcCer and GlcSph has been demonstrated to increase the risk of α-syn aggregation [116, 117]. While GlcCer and its downstream ganglioside derivatives are essential for nervous system development and synaptic function under physiological conditions [118, 119], their dysregulation represents a key pathogenic event.

The search for a consistent lipid profile in *GBA1*-PD has yielded conflicting results in both central nervous system tissues and biofluids. Recent research has reported elevated GlcSph levels across multiple brain regions in patients with *GBA1*-PD and dementia, with these levels correlating positively with phosphorylated α-syn [93]. However, a non-targeted lipidomics study found no significant increase in GlcCer in most brain regions of *GBA1*-PD patients, reporting instead an upregulation of downstream gangliosides levels (e.g., GM1 and GD1a/b) in the striatum, occipital cortex, and middle temporal gyrus [113]. The results from CSF are equally complex. Several studies suggest significantly elevated GlcCer and reduced Cer levels in *GBA1*-PD patients, which correlate with the severity of the *GBA1* mutation [85]. In contrast, another study found no significant differences in the CSF levels of GlcCer or GlcSph when comparing *GBA1*-PD to non-carrier PD and DLB patients [114]. This heterogeneity may arise from the differential susceptibility of distinct lipid subtypes to various *GBA1* mutations, disease stages, or the presence of comorbid dementia.

A significant analytical challenge complicates these findings: the presence of galactosylceramide (GalCer), a structural isomer of GlcCer. GalCer is a critical component of the myelin sheath and is nearly 10-fold more abundant than GlcCer in the brain and CSF [120]. Therefore, analytical protocols that do not employ chromatographic separation may lead to inaccurate quantification of GlcCer due to interference from the much higher abundance of GalCer. Although absolute GalCer levels appear unaltered in the brain tissue of *GBA1*-PD patients [93], recent studies have reported elevated GlcCer-to-GalCer median ratios in the CSF of these patients [121].

### Ceramide

As a direct downstream metabolite of GCase, Cer is fundamentally linked to the *GBA1* pathway. However, this well-established biochemical relationship has not translated into a clear clinical biomarker, as studies measuring Cer levels in *GBA1*-PD patients have yielded inconsistent and often contradictory results. The reported changes in Cer are highly variable, depending on the specific Cer species detected, the biological sample type, and the patient cohort. In the brain tissue of *GBA1*-PD patients, heightened activity of ceramide synthases 2 and 4 (CERS2 and CERS4) has been shown to promote the accumulation of long-chain C20- and C24-Cers [90]. Similarly, studies on peripheral biofluids have reported significantly elevated total Cer and SM concentrations in the serum [14]. Findings from CSF are more complex. While total Cer levels may not differ significantly from those in iPD, patients carrying severe *GBA1* mutations exhibit increased Cer levels [85], suggesting a mutation-specific effect.

GCase dysfunction can also impair Cer metabolism, specifically by reducing the synthesis of C18-Cer. This particular deficiency has been shown to disrupt the localization of the Rab8a protein, leading to secretory pathway defects, impaired autophagy, and ultimately, the exacerbation of α-syn aggregation [122]. A major challenge in establishing Cer as a specific biomarker for *GBA1*-PD is that its metabolism is also dysregulated in iPD. For instance, elevated plasma concentrations of C16:0 and C18:0 Cers have been found in iPD patients, where they correlate with cognitive impairment [123]. These findings collectively indicate that Cer species may not yet serve as reliable, specific biomarkers for *GBA1*-PD patients.

Despite its limitations as a biomarker, modulating the Cer pathway remains a viable therapeutic strategy. Studies have shown that inhibiting acid ceramidase (AC), the enzyme that converts Cer to sphingosine, with compounds like carmofur, can elevate Cer and GlcSph levels, leading to the improvement of α-syn pathology in GCase-deficient cellular models [122].

### Lipid droplets and cholesterol

Lipid droplets (LDs), dynamic intracellular organelles responsible for storing neutral lipids (e.g., triglycerides and cholesteryl esters), play critical roles in lipid homeostasis regulation [124]. Studies demonstrate that the overexpression of α-syn in cells leads to the accumulation of LDs and increased levels of triglycerides and cholesteryl esters [125]. Conversely, pharmacological inhibition of LD formation in yeast models exacerbates α-syn cytotoxicity, suggesting a cytoprotective role of LDs against α-syn toxicity [126]. Recent studies have found a significant increase in LD numbers within SH-SY5Y cells overexpressing the G*BA1* E326K variant; this effect is further potentiated by oleic acid treatment independently of changes in GCase activity [127]. In murine models of GD, pharmacological inhibition of GCase robustly upregulates the expression of perilipin genes (encoding key LD-associated proteins), thereby establishing a direct mechanistic link between GCase dysfunction and dysregulated LD metabolism [128].

Cholesterol is essential for maintaining membrane integrity, fluidity, and signal transduction. *GBA1* deficiency disrupts lysosomal function, leading to abnormal cholesterol accumulation. Lysosomal cholesterol accumulation has been observed in both *GBA1*-knockdown SH-SY5Y cells and mouse primary neurons treated with β- glucocerebrosidase inhibitors [129]. Furthermore, fibroblasts derived from *GBA1*-PD patients harboring the N370S mutation exhibit intracellular cholesterol accumulation and multilamellar body formation, potentially linked to compromised lysosomal membrane stability [130]. Such lysosomal cholesterol buildup may perturb cholesterol distribution within lipid rafts, thereby modulating α-syn-cholesterol interactions and accelerating α-syn oligomerization and pathological aggregation [131]. Notably, total cholesterol levels remain unaltered in putaminal and cerebellar tissues obtained from *GBA1*-PD patients [132]. However, findings regarding serum cholesterol are conflicting: some studies report reduced total cholesterol and low-density lipoprotein levels in *GBA1*-PD patients [133], while others show no significant differences compared to controls [14]. These discrepancies suggest that cholesterol dysregulation may be tissue-specific or may be compensated by other mechanisms, highlighting the need for further research to elucidate its precise pathological significance.

## Potential biomarkers of *GBA1*-PD

Multi-omics profiling of clinically relevant biospecimens, particularly peripheral blood and CSF, from *GBA1*-PD patients has uncovered a panel of candidate biomarkers (Tables 1, 2 and 3). Among them, GCase activity exhibits the most direct association with *GBA1* mutations. Compared to iPD, GCase activity is consistently and significantly reduced in blood, CSF and brain tissue of *GBA1*-PD patients. Moreover, the extent of this reduction correlates with mutation severity and represents a biomarker-associated risk factor for dementia in PD [85, 86, 88]. Nevertheless, variables such as sample type, cellular composition, and methodological variations can profoundly influence experimental outcomes [91]. Therefore, these factors must be meticulously controlled in both experimental design and clinical applications to ensure the reliability of GCase activity as a biomarker. Previous reports indicated that plasma exosomal α-syn levels are significantly higher in PD patients than in healthy controls [103]. More specifically, the ratio of exosomal α-syn to total α-syn in plasma EVs was found to be elevated in PD and negatively correlated with disease severity and GCase activity, directly linking the biomarker to the *GBA1* pathway [134]. However, recent studies report that while both *GBA1*-PD and iPD patients show higher plasma exosomal α-syn levels than healthy controls, there is no significant difference between the two PD groups [95]. These divergent findings highlight the need for further investigation to clarify the role of exosome-mediated α-syn regulation in the pathogenesis of *GBA1*-PD and to validate its potential as a targeted biomarker.

**Table 3:** Proteomics profiling and associated dysregulated pathways in *GBA1*-PD patients

Proteins	Samples	Proteinomics methods	Sample type	Associated Category	Reference
GCase	*GBA1*-PD (*n* = 21) iPD (*n* = 21) Control (*n* = 21)	Non-targeted, mass spectrometry based quantitative discovery proteomics	Post-mortem Human Brain Tissue	sphingolipid metabolism	Blumenreich S et al. [90]
ADI1				methionine metabolism	
MAPT, TUBB2B, MAP1B, MAP6, NRCAM	*GBA1*-PD (*n* = 3) Control (*n* = 4)	Non-modified Proteome	*GBA*-PD iPSC-dopamine neurons	microtubule dynamics, neurite outgrowth, and axon extension	Bogetofte H et al. [108]
APOE				Alzheimer disease	
mTOR		Phosphoproteome		mTOR signaling pathway	
LAMP1, LAMP2, cathepsin D		Sialylated Glycoproteome		lysosomal proteins	
ASAH 1, GALC, PSAP, GLA				sphingolipid metabolism	
CNTN1, SELENBP1, aGPCRs, APOE	*GBA1*-PD (*n* = 22) iPD (*n* = 7) Control (*n* = 3)	SWATH-mass spectrometry	CSF	synaptic damage, oxidative stress, neuronal loss, and α-syn aggregation	Zafar S et al. [109]
FKBP4	*GBA1*-PD (*n* = 17) iPD (*n* = 17)	Proximity Extension Assay	CSF	the regulation of steroid receptor activity	Kojima R et al. [110]

Gcase: acid-β-glucosidase; ADI1: 1,2-dihydroxy-3-keto-5methylthiopentene dioxygenase; MAPT: microtubule-associated protein tau; TUBB2B: tubulin b-2B chain; NRCAM: neuronal cell adhesion molecule; APOE: apolipoprotein E; mTOR: mammalian target of rapamycin; LAMP1: lysosomal-associated membrane protein 1; LAMP2: lysosomal-associated membrane protein 2; ASAH 1: acid ceramidase; GALC: galactocerebrosidase; PSAP: prosaposin; GLA: galactosidase A; CNTN1: Contactin-1; SELENBP1: Selenium-binding protein 1; aGPCRs: Adhesion G Protein-Coupled Receptor; FKBP4: FK506-binding protein 4

High-throughput proteomic, transcriptomic, and lipidomic analyses have identified potential biomarkers capable of differentiating *GBA1*-PD from iPD. Specifically, proteomic analysis of CSF revealed that the levels of CNTN1, SELENBP1, GPCRs, and APOE were significantly reduced in *GBA1*-PD patients compared to both iPD patients and healthy controls, whereas no significant differences were observed between iPD patients and controls [109]. Moreover, elevated levels of FKBP4 were recently reported in both the CSF and cell culture supernatant of iPSC-derived midbrain dopaminergic neurons from *GBA1*-PD patients [110]. Furthermore, comparisons between symptomatic *GBA1*-PD patients and asymptomatic carriers indicate that clinical conversion is associated with the significant deregulation of genes involved in lysosomal function, membrane trafficking, and mitochondrial processing. The downregulation of *DUSP1* and *COLEC12*, and the upregulation of *TRIM13* and *BCL6*, have been identified as potential biomarkers to distinguish symptomatic patients from asymptomatic carriers [81]. However, defining a clear biomarker signature based on specific lipid species remains challenging, as reported changes are often inconsistent across studies and overlap significantly with alterations observed in iPD [123]. Recent research suggests that the ratios of specific lipid species may serve as more robust biomarkers. It has been reported that GlcCer-to-GalCer median ratios were higher in the CSF of *GBA1*-PD patients (particularly those with severe *GBA1* mutations) than in iPD and healthy controls [121]. Similarly, an elevated CSF GlcCer/SM ratio has been proposed as a potential prognostic marker for cognitive decline in *GBA1*-PD [135]. Future studies employing rigorous and standardized analytical methods are necessary to conclusively determine whether these lipid ratios can serve as reliable biomarkers for *GBA1*-PD.

Identifying the prodromal stage of PD remains a substantial challenge for early diagnosis. Compared to patients with iPD and *LRRK2* carriers, the prevalence of RBD is significantly elevated among *GBA1* mutation carriers, particularly those harboring severe mutations [46, 136]. Moreover, even during the asymptomatic phase, *GBA1* carriers exhibit more pronounced cognitive decline and heightened anxiety than non-carriers [47]. A cross-sectional study using α-syn seed amplification assays (SAA) in the Parkinson’s Progression Markers Initiative (PPMI) cohort indicated that the assay classifies PD with high sensitivity and specificity, and detects prodromal individuals (e.g., those with RBD and hyposmia) prior to clinical diagnosis [137]. However, these prodromal features alone are insufficient to definitively identify those who will eventually develop PD. Therefore, future research utilizing multi-omics approaches to characterize the dynamic alterations of genes, proteins, and metabolites in *GBA1* mutation carriers during the transition from the prodromal phase to PD may offer novel insights into the molecular pathogenesis of *GBA1*-PD and facilitate early diagnosis.

## Conclusions and perspective

Multi-omics investigations have profoundly advanced our understanding of *GBA1*-PD, establishing it as a distinct, genetically defined subtype of synucleinopathy with unique pathobiological signatures across molecular layers. It is a systemic disease characterized by a core failure of the lysosomal-lipid-protein homeostasis network. This primary pathology is exacerbated by concomitant mitochondrial dysfunction, impaired autophagic flux, and sustained neuroinflammation, which collectively drive an aggressive neurodegenerative phenotype.

Despite this progress, significant challenges remain. The extensive molecular overlap between *GBA1*-PD and iPD, coupled with a reliance on single-omics analysis, limits the identification of disease-specific markers. Therefore, longitudinal multi-omics studies are essential for future research. The integration of complementary omics data will facilitate a systemic and comprehensive understanding of *GBA1*-PD heterogeneity, allowing researchers to capture the complex interplay between genes, proteins, and metabolites. By monitoring these signatures across different disease stages, such research holds the potential to yield robust biomarkers for early diagnosis and prognosis. Additionally, current findings require stringent validation, as discrepancies often arise from clinical sample heterogeneity (e.g., disease progression, lifestyle, diet), preanalytical variables (such as freeze-thaw cycles), and methodological differences in analytical workflows. A key priority for future research is the establishment of standardized analytical pipelines coupled with precise patient stratification based on criteria such as *GBA1* mutation type and severity. Such a framework is essential for uncovering the complex heterogeneity of *GBA1*-PD, which will, in turn, facilitate the discovery of biomarkers for precision diagnostics and the development of targeted therapeutics.

## Data Availability

No datasets were generated or analysed during the current study.

## Abbreviations

α-syn: α-synuclein
aGPCRs: Adhesion G protein-coupled receptors
APOE: Apolipoprotein E
ALP: Autophagy-lysosomal pathway
ADI1: Acireductone dioxygenase 1
CSF: Cerebrospinal fluid
Cer: Ceramide
CNTN1: Contactin-1
DLB: Dementia with Lewy bodies
DTI: Diffusion tensor imaging
DAT: Dopamine transporter density
ER: Endoplasmic reticulum
EVs: Extracellular vesicles
FDC: Fiber density and cross section
GBA1-PD: GBA1-associated Parkinson’s disease
GD: Gaucher disease
GCase: β-glucocerebrosidase
GlcCer: Glucosylceramide
GlcSph: Glucosylsphingosine
GalCer: Galactosylceramide
iPD: Idiopathic PD
iPSCs: Induced pluripotent stem cells
LacCer: Lactosylceramide
LDs: Lipid droplets
MRI: Magnetic resonance imaging
OR: Odds ratios
PD: Parkinson’s disease
PET: Positron emission tomography
PBMCs: Peripheral blood mononuclear cells
RBD: Rapid eye movement sleep behavior disorder
SCD: Stearoyl-CoA desaturase
SPECT: Single-photon emission computed tomography
SUVRs: Standardized uptake value ratios
SELENBP1: Selenium-binding protein 1
UPR: Unfolded protein response
WM: White matter
